# Intracellular chloride concentration of the mouse vomeronasal neuron

**DOI:** 10.1186/s12868-015-0230-y

**Published:** 2015-12-15

**Authors:** SangSeong Kim, Limei Ma, Jay Unruh, Sean McKinney, C. Ron Yu

**Affiliations:** Stowers Institute for Medical Research, 1000 East 50th Street, Kansas City, MO 64110 USA; Department of Pharmacy, Institute of Pharmaceutical Science and Technology, Hanyang University, Seoul, Gyeonggi-do Republic of Korea; Department of Anatomy and Cell Biology, University of Kansas Medical Center, Kansas City, KS 66160 USA

**Keywords:** Vomeronasal organ, TRPC2, Pheromones, Calcium activated chloride channel

## Abstract

**Background:**

The vomeronasal organ (VNO) is specialized in detecting pheromone and heterospecific cues in the environment. Recent studies demonstrate the involvement of multiple ion channels in VNO signal transduction, including the calcium-activated chloride channels (CACCs). Opening of CACCs appears to result in activation of VNO neuron through outflow of Cl^−^ ions. However, the intracellular Cl^−^ concentration remains undetermined.

**Results:**

We used the chloride ion quenching dye, MQAE, to measure the intracellular Cl^−^ concentration of VNO neuron in live VNO slices. The resting Cl^−^ concentration in the VNO neurons is measured at 84.73 mM. Urine activation of the VNO neurons causes a drop in Cl^−^ concentration, consistent with the notion of an efflux of Cl^−^ to depolarize the cells. Similar observation is made for VNO neurons from mice with deletion of the transient receptor potential canonical channel 2 (TRPC2), which have a resting Cl^−^ concentrations at 81 mM.

**Conclusions:**

The VNO neurons rest at high intracellular Cl^−^ concentration, which can lead to depolarization of the cell when chloride channels open. These results also provide additional support of TRPC2-independent pathway of VNO activation.

## Background

Chloride ion plays an important role in controlling cell excitability. Cl^−^ flowing through GABA receptors is generally inhibitory, but in developing brain, high intracellular Cl^−^ concentration allows the efflux of Cl^−^ making it excitatory. Likewise, the mammalian olfactory sensory neurons maintain an intracellular Cl^−^ concentration of ~69 mM [1] or 40–50 mM [2]. Odor stimulation activates the cyclic nucleotide-gated (CNG) channel and results in calcium influx activating calcium-activated chloride channels (CACCs) to depolarize the neurons [3–10].

Recent studies have revealed the involvement of a chloride conductance in VNO activation. In VNO neuron, urinary stimulus appears to activate CACC currents. The chloride conductance is carried by TMEM16A/anoctamin1 [11] and can be activated independently of the TRPC2 channel, therefore constitutes a parallel signal transduction pathway [12–14]. The level of intracellular Cl^−^ is critical in determining the direction of current flow. With high [Cl^−^]_in_, efflux of Cl^−^ would result in an inward current to depolarize the cells. Conversely, at low [Cl^−^]_in_, the ions may not flow across the cell membrane or flow intracellularly leading to an outward current to hyperpolarize the cells. In slice and single cell recordings [12–14], activation of CACC currents appears to depolarize the VNO neurons, suggesting that these cells maintain a high intracellular Cl^−^ concentration. However, the concentration of Cl^−^ in VNO neurons has not been determined.

We sought to determine the intracellular Cl^−^ concentration of VNO neurons in live slice preparations. In the olfactory neurons, intracellular ion concentrations were first determined by energy-dispersive X-ray microanalysis in cryosections of the rat olfactory epithelium [1]. This method required highly specialized equipment that is not easily accessible. Kaneko and colleagues measured intracellular Cl^−^ concentration in the olfactory neuron by using *N*-[ethoxycarbonylmethyl]-6-methoxy-quinolinium bromide (MQAE) as a fluorescent chloride ion indicator [2, 15]. In this study we adopt this method to measure intracellular Cl^−^ concentration in the mouse VNO neurons.

## Results and discussion

### Intracellular Cl^−^ concentration in the VNO neurons

**Fig. 1:**
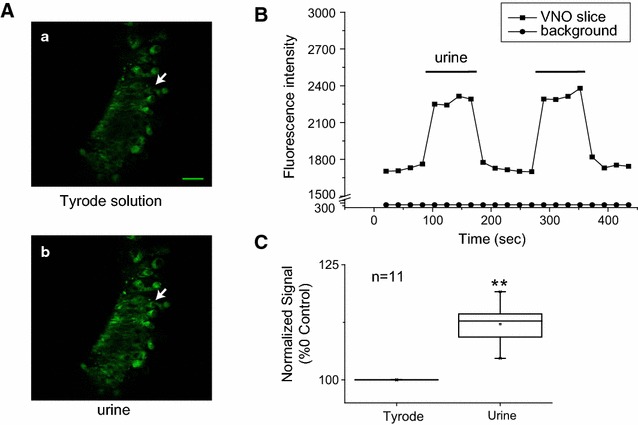
Urine induced change in MQAE fluorescence in VNO slices. **A** Image of VNO slice in Tyrode solution (*a*) and in response to urine stimulation (*b*). *Scale bar* is 25 μm. **B** Quantitative measurement of fluorescence signal during urine application for the cell indicated by the *arrows*. *Square* indicates VNO slice image and circle for background change. **C** *Box plot* indicates relative fluorescence changes in response to urine (1.12 ± 0.01, n = 11). *Box plots* show the mean (*central point*), median (*central horizontal line* in the *box*), maximal and minimal values (*short horizontal lines*), 99 and 1 % range (*crosses* at both ends of the *box*), 5–95 % percentile range (*whiskers*) and 25–75 % range (*box*). **P < 0.05, Student’s t test

MQAE is used as a chloride fluorescent indicator to determine the intracellular Cl^−^ concentration because of its collisional quenching property. High Cl^−^ concentration quenches its fluorescent signal. It has been successfully used to monitor the intracellular Cl^−^ concentrations of olfactory neurons, the dorsal roots ganglion cells, salivary glands as well as brain slices [2, 15, 16, 17, 18]. We prepared VNO slice at 100 μm thickness to preserve the intact neuronal structure including the dendrite. Incubating VNO slice with MQAE for 30 min resulted in robust fluorescence in the VNO neurons. After loading we measured the fluorescent signals in the VNO neurons incubated in Tyrode solution before and after urine application (Fig. 1). Fluorescent signals in VNO slice noticeably increased during urine application, indicating a reduction of intracellular Cl^−^. The elevation of fluorescent signals lasted the entire duration of urine application. This is consistent with previous observation that the VNO neurons exhibit non-adaptive responses [19, 20]. Over the course of urine application there is no change in background fluorescence, indicating that the changes were not from signals of urine application (Fig. 1B). On average there was a 12 % increase in fluorescence (P < 10^−14^; n = 11). This observation is consistent with previous observation that urine induces efflux of Cl^−^ from VNO neurons [12–14].

To estimate the intracellular Cl^−^ in slice preparation, we measured the fluorescent signals in a series of standard Cl^−^ solutions. The neurons were permeabilized with ionophores and incubated with the standard solutions. Cl^−^ homeostasis is maintained by chloride co-transporters including Na–K–Cl co-transporter [21, 22] and K–Cl co-transporter [23]. The homeostasis mechanism may skew the results when the standard solutions dramatically alter the intracellular salt balance. Thus, in addition to chloride ionophore 1, we used the Cl^−^/OH^−^ ionophore tributyltin to remove transmembrane H^+^/OH^−^ gradients. We also include nigericin, an ionophore for H^+^ , K^+^ and Cl^−^, to remove the contribution of cations in resetting Cl^−^ gradient. A previous study has shown that these ionophores dissipate Cl^−^ gradients across the plasma membrane [24]. By abolishing Cl^−^ gradient, the intracellular Cl^−^ concentration could be equilibrated with the extracellular Cl^−^.

**Fig. 2:**
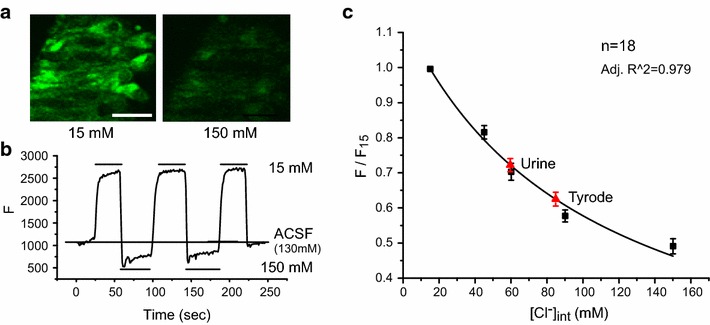
Measurement of intracellular chloride concentration in VNO neuron. **a** Fluorescence signal of a VNO slice loaded with MQAE dye and incubated in ionophore cocktail in 15 mM (*left*) and 150 mM (*right*) standard solutions. *Scale bar* is 10 μm. **b** Repeated calibration using the two standard solutions indicates no obvious bleaching. Fluorescent intensity (arbitrary unit) is plotted over time. Top three *bars* indicate incubation with 15 mM solution and bottom two *bars* indicate incubation with 150 mM solution. ACSF is used before and after the calibration. **c** Stern–Volmer equation fit (*black lines*) to fluorescent signals using standard intracellular chloride concentration (*black squares*). Average intensities of MQAE fluorescence of VNO neurons at rest (Tyrode) and during urine application are extrapolated to obtain [Cl^−^]_in_. Data is shown as mean ± s.e.m

Following measurements before and after urine application without ionophores, we measured MQAE fluorescence by incubating the VNO slices with standard solutions containing the ionophore cocktail. MQAE fluorescent intensity was high at low [Cl^−^] and the signal was quenched at high [Cl^−^] (Fig. 2a). Changing external [Cl^−^] led to quick changes of intracellular fluorescent signals in the slices. We performed repeated imaging using 15 and 150 mM standard solutions over a period of 4 min. The fluorescent intensity at 15 mM [Cl^−^] reached the same levels during three applications (Fig. 2b). There was no bleaching effect as observed in previous studies [2]. The difference may be explained by increased sensitivity of a new generation of confocal microscope, which required less laser power to excite the fluorophores.

We applied standard solutions containing 15, 45, 60, 90 or 150 mM Cl^−^ and measured fluorescent intensities. All measurements from the same cell were normalized to the highest fluorescent intensity, measured with 15 mM standard chloride solution (Fig. 2c). The data were fit with the Stern–Volmer equation with linear regression. We used fluorescent signals measured at rest and during urine stimulation to extrapolate [Cl^−^]_in_ using the standard curve. The intracellular Cl^−^ concentration was determined to be 84.73 ± 2.65 mM (Fig. 2c; mean ± SD) in tyrode solution. Urine-activation caused detectable increase in fluorescence, indicating a drop in intracellular Cl^−^ concentration resulting from the efflux of Cl^−^ ions (Fig. 1). With the same method the intracellular Cl^−^ concentration in urine presence was calculated as 59.5 ± 1.54 mM, which was 25.23 mM reduction from resting condition (Fig. 2c).

### Cl^−^ concentration of VNO neuron in TRPC2−/− mice

**Fig. 3:**
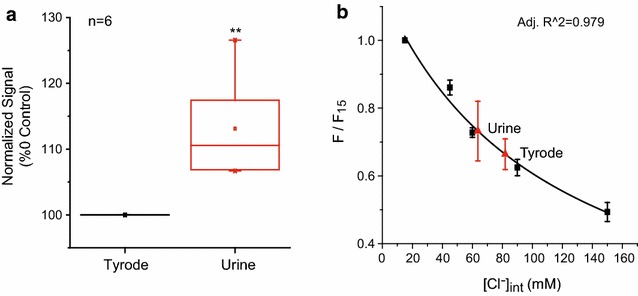
Measurement of intracellular chloride concentration in TRPC2 mutant VNO neuron. **a** *Box plot* indicates the relative fluorescence level change in response to urine application (1.13 ± 0.03, n = 6). **b** Stern–Volmer equation fit (*black line*) to fluorescent signals using standard intracellular chloride concentration (*black squares*) yield Ksv = 8.89 × 10^−3^ mM^−1^ (n = 6). MQAE fluorescence of VNO neurons at rest (Tyrode) and during urine application are extrapolated to determine [Cl]_int_ (*red triangles*). *Box plots* show the mean (*central point*), median (*central horizontal line* in the *box*), maximal and minimal values (*short horizontal lines*), 99 and 1 % range (*crosses* at both ends of the *box*), 5–95 % percentile range (*whiskers*) and 25–75 % range (*box*). **P < 0.05, student’s t test. Data are shown as mean ± s.e.m

In previous studies, we have identified independent pathways in activating the VNO [13, 14]. Although Ca^2+^ entry through the TRPC2 channels contribute to the activation of CACC, Ca^2+^ mobilized from the intracellular store can also activate CACC in the absence of TRPC2. We carried out the same experiments in TRPC2−/− VNO slices to measure [Cl^−^]_in_. In TRPC2−/− VNO, urine elicited an average of 13 % increase in fluorescence (Fig. 3a, p < 10^−6^, n = 6), indicating that urine elicited a drop in [Cl^−^]_in_. We then calibrated MQAE signals using standard solutions. Linear regression fit from the TRPC2−/− VNO (Fig. 3b) generated similar values of Stern–Volmer constant (8.79 × 10^−3^ mM^−1^ in wild type and 8.89 × 10^−3^ mM^−1^ in TRPC2−/−). [Cl^−^]_in_ measured at resting state was 81.89 ± 5.59 mM, nearly the same as the wildtype (Fig. 3b). Urine application led to an increase in fluorescence, which corresponded to a decrease in [Cl^−^]_in_ to 63.65 ± 7.65 mM. The concentration reduction was 18.24 mM. The similarity of measured [Cl^−^]_in_ between wildtype and TRPC2−/− samples is consistent with our previous finding of a TRPC2 independent pathway in activating the chloride conductance [13, 14].

## Conclusions

The vomeronasal organ is involved in detection of pheromone cues, which are contained in bodily fluids and excretions. Pheromone-containing fluids, including urine, contain varying salt concentrations that may affect pheromone detection. The vomeronasal organ appears to have evolved specific mechanisms in mitigating the impact by the large variation of ionic concentrations. For example, the supporting cells have specialized ion conductance for K^+^ and Cl^−^ [25]. Recent studies have shown that the VNO utilizes different signal transduction pathways in parallel to mediate its activation [12, 13, 14, 26, 27, 28]. Existing evidence suggests that CACC is involved in VNO signal transduction [12–14]. The ion channels responsible for Cl^−^ conductance is likely to be anoctomin 1 (TMEM16A) and anotamin 2 (TMEM16B), which have been found to be located in the dendritic knob of the VNO sensory neurons [27]. The latest study show that CACC is abolished in the VNO of mice without TMEM16A [11]. In this study, we determine [Cl^−^]_in_ for VNO neurons at 84.73 ± 2.65 mM for wildtype and 81.89 ± 5.59 mM for TRPC2−/− mice. The values we obtain are remarkably similar to what is observed in the olfactory sensory neurons [1, 2, 15, 29]. Under conditions in which electrophysiology and imaging experiments are conducted (external [Cl^−^] at ~130 mM), the reversal potential for Cl^−^ calculated from Nernst Equation is at −21 mV. Because VNO neurons rest at ~−50 mV, the activation of chloride channels would lead to an outward flow of Cl^−^, causing depolarization of the neurons. Consistent with the notion that the CACC can be activated independently from TRPC2, urine application elicited changes in fluorescent signals in TRPC2−/− VNO. It is noticeable that the resting [Cl^−^]_in_ in TRPC2−/− VNO is slightly lower than the wildtype. Moreover, urine induced change of [Cl^−^]_in_ is slightly smaller in TRPC2−/− than in wild type, although the difference is not statistically significant. This evidence provides further support of parallel pathways of VNO activation. It also indicate that TRPC2 may contribute to regulate basal levels of [Cl^−^]_in_ and to influx of Cl^−^. A reduction in Ca^2+^ influx may reduce CACC activation.

We note that our measurements are from VNO cell body with intact dendrite. Although we observe concomitant change in fluorescent intensity in the dendrites as the cell bodies, technical challenges of imaging in slice have prevented us from accurately track individual dendritic structure. Because intracellular changes are observed in both dendrites and cell bodies during pheromone stimulation, it is likely that calcium activated channels are present in both compartments. Although TMEM16A and TMEM16B may be concentrated in the dendrite to mediate primary response, the expression of bestrophin 2 may allow Cl^−^ movement in the cell body [12, 28].

## Methods

### Animals

Pheromone-evoked responses were obtained from a total seven of 2–6 months old mice of the C57BL/6 J strain and one TRPC2−/− mouse of same background. Approximately equal numbers of male and female mice were used. Animals were maintained in the Lab Animal Service Facility of Stowers Institute at 12:12 light cycle, and provided with food and water ad libitum. Experimental protocols were approved by the Institutional Animal Care and Use Committee at Stowers Institute and were in compliance with NIH Guide for Care and Use of Animals. Urine samples were collected from mature male and female C57BL/6J animals using the free-catch method. Equal volumes of male and female urine were mixed and diluted to 1:100 in Ringer’s solution for stimulation.

### Slice preparation

Mice were sacrificed by rapid decapitation after CO_2_ asphyxiation and the VNOs were dissected out into mouse artificial cerebrospinal fluid (mACSF) that was continuously bubbled with 5 % CO_2_/95 % O_2_ and maintained at 4 °C. The tissue was embedded in a gel composed of 4 % low melting point agarose dissolved in mACSF at 37 °C, chilled on ice, mounted on a specimen tray, and secured onto the VF-300 microtome sectioning system (Precisionary Instruments). Tissue samples were sectioned into 100 μm slices, which were then transferred to mACSF solution and continuously bubbled with 5 % CO_2_/95 % O_2_ at room temperature. The composition of mACSF is (in mM): NaCl 125, KCl 2.5, CaCl_2_ 2, MgCl_2_ 1, NaHCO_3_ 25, Na_2_HPO_4_ 1.25, Glucose (Dextrose) 10.

### Measurement of intracellular Cl^−^

VNO slices were incubated with 5 mM *N*-(ethoxycarbonylmethyl)-6-methoxyquinolinium bromide (MQAE, molecular probes) for 30 min at room temperature in Tyrode solution before it was transferred to a perfusion chamber for imaging using a Zeiss AxioSkope FS2 microscope with a 40× water-dipping lens (0.8 N.A; 3.3 mm working distance). Tyrode solution is composed of (in mM): 140 NaCl, 5 KCl, 2 CaCl_2_, 1 MgCl_2_, 10 glucose, and 10 HEPES, pH 7.2. MQAE was excited with 2-photon 750 nm laser generated from the Chameleon XR system (Coherent). Z-stack images of the VNO slice were recorded with 3 μm step increment for each condition. VNO slices were imaged in Tyrode solution and during urine application.

Urine stimulation and standard solutions are delivered using a perfusion system that has been described previously [30, 31]. The system is under constant pressure and the switching between different solutions does not affect the flow rate and does not cause mechanical movement of the slices. Following urine application, the slices were treated with a series of standard Tyrode solution containing different predetermined concentrations of Cl^−^ (15, 45, 60, 90 and 150 mM). The standard solutions were applied together with the ionophore cocktail to calibrate fluorescent signals. The calibration solutions contain the following chemicals: 5 μM Cl^−^/OH^−^ antiporter tributyltin (Sigma), 3.5 μM K^+^/H^+^ antiporter nigericin (Sigma) and 1.5 μM chloride ionophore 1 (GFS chemicals). Calibration solution consisted of (in mM) 150 KCl, 2 CaCl_2_, 10 glucose and 10 HEPES (pH 7.2), with different concentrations of KCl replaced with equal molar KNO_3_ to achieve desired extracellular Cl^−^ concentrations. Between applications of each different concentration solutions, the sample was washed with Tyrode solution. During imaging, excitation light with 15 s interval was applied to prevent from bleaching of MQAE.

Individual images were analyzed to measure the intensity of MQAE in selected region of interest (ROI). In previous studies, we have shown that approximately 30 % of cells in slice preparations respond to urine stimulation [13, 14, 32, 19]. We have selected 11 cells with responses to urine stimulation as ROI to measure the response to urine. Additional 7 cells are also selected to measure basal level of [Cl^−^]. Because it is difficult to assign the dendritic knob to a specific cell in slice preparations, we only focused on the cell bodies. All analyses were performed off-line. Individual cells in the VNO slices may shift out of focus. To solve this problem, we took Z-stacks of the slice and track individual cells in different Z-plane. Cells with intact morphology that can be discerned during the entire imaging process were used for intensity analysis. ROI of 18 neurons from 7 different slices were selected for analyses. In case of TRPC2−/− mice imaging, a single slice was used. Intensity values measured in each calibration solution were normalized to that of the initial 15 mM Cl^−^ standard solution within individual VNO slices. The averaged data points were fitted using the Stern–Volmer equation [15]:

1 $$F_{a} = \frac{{F_{0} }}{{1 + K_{q} [Cl^{ - } ]_{in} }}$$

where F_a_ stands for intensity at a given Cl^−^ concentration, F_0_ for intensity at zero chloride and K_q_ for a quenching constant. By inserting each value of F_a_, F_0_, and [Cl^−^]_in_ into the formula, Kq at VNO neuronal cell body is calculated to be 8.79 × 10^−3^ mM^−1^ in wild type and 8.89 × 10^−3^ mM^−1^ in TRPC2−/−. The relative values of MQAE fluorescence (normalized to 15 mM Cl^−^) measured in Tyrode solution and during urine application were averaged to extrapolate specific intracellular Cl^−^ concentrations from the standard curve.

## Abbreviations

VNO: The vomeronasal organ
TRPC2: Transient receptor potential canonical channel 2
CACC: Calcium-activated chloride channels
CNG: Cyclic nucleotide gated
MQAE: *N*-[ethoxycarbonylmethyl]-6-methoxy-quinolinium bromide
mACSF: Mouse artificial cerebrospinal fluid
